# Safety and Efficacy of Mesenchymal Stem Cells for the Treatment of Evolving and Established Bronchopulmonary Dysplasia: A Systematic Literature Review

**DOI:** 10.7759/cureus.32598

**Published:** 2022-12-16

**Authors:** Sheiniz Giva, Ahmed Abdelrahim, Blessing T Ojinna, Venkata Pravallika Putrevu, Elisa A Bornemann, Hadi Farhat, Kavya Amaravadi, Mahmoud Ben Abdallah, Sai Dheeraj Gutlapalli, Sai Sri Penumetcha

**Affiliations:** 1 Neonatology, Temple University Hospital, Dublin, IRL; 2 Research, California Institute of Behavioral Neurosciences & Psychology, Fairfield, USA; 3 Internal Medicine, Beaumont Hospital, Michigan, USA; 4 General Medicine, University of Nigeria Nsukka, College of Medicine, Enugu, NGA; 5 Internal Medicine, Neurostar Multi-speciality Hospital, Kakinada, IND; 6 Internal Medicine, University of Balamand, Beirut, LBN; 7 Internal Medicine, Manchester University NHS Foundation Trust, Manchester, GBR; 8 General Medicine, Chalmeda Anand Rao Institute of Medical Sciences, Karimnagar, IND

**Keywords:** respiratory distress syndrome of prematurity, mesenchymal stem cells, stem cells, chronic lung disease of prematurity, bronchopulmonary dysplasia

## Abstract

Bronchopulmonary dysplasia (BPD) is a frequent sequela of modern medicine when infants are born prematurely. Currently, there is no single treatment or combination of treatments to prevent or fully treat BPD. Mesenchymal stem cells (MSCs) have promising properties that could aid in the reversal of lung injury, as seen in patients with BPD. This study reviews the available evidence regarding the safety and efficacy of the use of MSCs for the treatment of evolving and established BPD. This systematic review was performed in accordance with the Preferred Reporting Items for Systematic Reviews and Meta-Analyses (PRISMA). We found eight studies that fulfilled the inclusion and exclusion criteria. While all studies proved the safety and efficacy of MSCs administered intravenously and intratracheally, the only available randomized controlled trial (RCT) failed to demonstrate the benefit of MSC administration in the early treatment of BPD. The remaining studies varied between phase I clinical trials and case reports, but all seemed to show some evidence that MSCs may be of benefit in the late treatment of established BPD. Considering some of the studies have less evidence, early treatment to prevent lung fibrosis may be more successful, particularly in the younger gestational ages where lung development is more immature, and research should focus on this.

## Introduction and background

Bronchopulmonary dysplasia (BPD) is a chronic lung disease commonly seen in premature infants with respiratory distress syndrome, requiring mechanical ventilation and supplemental oxygen therapy [1]. It is estimated to have a prevalence of about 40% to 50% of infants born at <28 weeks of gestation and up to 80% of infants born at <24 weeks of gestation [2,3].

Bronchopulmonary dysplasia, initially described in 1967 by Northway et al. [4], was thought to be caused by inflammatory and fibrotic changes leading to the remodelling of lung parenchyma secondary due to high mechanical pressures and oxygen concentrations [5,6]. While the primary cause of BPD is still exposure to elevated oxidative stress and mechanical stretch, it is now thought that other factors contribute to its development, such as exposure to infection and inflammation as well as poor nutritional state and growth restriction [1]. There is also the belief that genetics play a role in the development of BPD [1]. Over the years and with the birth and resuscitation of premature infants born at younger gestation ages, the definition of BPD has evolved. The current definition of BPD and its grading can be seen below in Table 1 [7].

**Table 1:** Definition of bronchopulmonary dysplasia (BPD)

Gestational Age	< 32 weeks at birth	> 32 weeks at birth
Time of assessment	36 weeks or discharge home	Between 28 and 56 days or discharge home
	Oxygen > 28 days PLUS	
Mild BPD	Self-ventilating on room air	
Moderate BPD	Need for < 30% oxygen	
Severe BPD	Need for > 30% oxygen or positive pressure	

Multiple strategies have been developed to prevent the development of BPD, such as gentler ventilation strategies from birth, exogenous surfactant administration, and caffeine [1,8]. Other pharmacotherapies have also been suggested and are currently in use (e.g., corticosteroids and diuretics). With the development of modern medicine, BPD management has become more challenging as infants are born with lower birth weight and gestational age, making their lungs more immature [9]. Despite all the research on the prevention and treatment of BPD, it remains the most frequent sequela of premature birth, with infants having a generally poor prognosis and requiring prolonged respiratory support [10,11].

Stem cells have the ability to self-differentiate and can be obtained from many different tissues [12,13]; cells obtained from foetal tissues seem to have greater proliferative capacity [9]. Mesenchymal stem cells (MSCs) are a type of stem cells that have previously been described as anti-inflammatory, proangiogenic, antifibrotic, and antioxidative [10] and have been routinely used in the treatment of disorders such as leukaemia and certain genetic conditions [13,14].

Mesenchymal stem cells are active cells that stimulate the production of growth and differentiation factors, promoting the endogenous repair processes of injured cells [12].

Multiple experimental animal models of BPD and lung injury have shown the positive effect of both intravenous and intratracheal administration of MSCs in reducing fibrosis and promoting the normal development of alveoli [15]. More recently, MSCs have been thought to be the key to providing the leap in research required for the treatment of established BPD [9]. Therefore, multiple trials are currently in place to assess the efficacy of this innovative strategy.

For this reason, a systematic literature review was conducted to evaluate the available data and assess the safety and preliminary efficacy of the administration of MSCs in premature infants with evolving or established BPD.

## Review

Methods

Protocol

This systematic review of the published literature was performed in accordance with the Preferred Reporting Items for Systematic Reviews and Meta-Analyses (PRISMA) [16]. Prior to selecting the final publications for analysis, a protocol was devised with strict inclusion and exclusion criteria and outcome measures. 

Inclusion and Exclusion Criteria

Studies eligible for analysis followed strict inclusion and exclusion criteria outlined below.

Inclusion criteria: Studies performed in very premature infants (born < 32 weeks of gestation) with established or evolving BPD (characterized as needing high levels of invasive respiratory support and respiratory deterioration in patients at increased risk of developing BPD); studies in which stem cells were used for the treatment of established BPD or deteriorating respiratory condition; randomized controlled trials (RCT), non-RCT and phase I safety and feasibility trials, cohort and case-control studies, case reports and case series.

Exclusion criteria: Studies published before January 2017; studies not published in English; experimental research studies in animals; studies in which the full text was not available for free; systematic reviews, meta-analysis, opinion, and editorials.

For this review, the accepted definition of BPD included both the old definition initially coined by Northway et al. in 1967 [4] ("28 days of oxygen exposure with characteristic radiographic changes") and the newer definition agreed upon by the National Institutes of Health (NIH) consensus in 2000 ("infants born < 32 weeks, requiring supplemental oxygen for at least 28 days and at 36 weeks postmenstrual age") [2].

Information Sources

The literature review was performed between July and August 2022 using four databases-PubMed, Medline, PubMed Control, Google Scholar-and also by reviewing reference lists of relevant publications.

Search

The database search included the following concepts and keywords outlined in Table 2.

**Table 2:** Database search MeSH: Medical subject headings

Concepts	Key words	MeSh strategy
Concept 1	Stem cells, Mesenchymal stem cells	"Stem Cells/therapy" OR "Stem Cells/transplantation"
Concept 2	Bronchopulmonary Dysplasia, Chronic Lung Disease of Prematurity	"Bronchopulmonary Dysplasia/drug therapy" OR "Bronchopulmonary Dysplasia/therapy"

The above search terms were input into the PubMed database as follows: (Stem cells OR Mesenchymal stem cells OR "Stem Cells/therapy"[Majr] OR "Stem Cells/transplantation"[Majr]) AND (Bronchopulmonary Dysplasia (BPD) OR Chronic Lung Disease of Prematurity OR "Bronchopulmonary Dysplasia/drug therapy"[Majr] OR "Bronchopulmonary Dysplasia/therapy"[Majr]).

Study Selection

**Figure 1:**
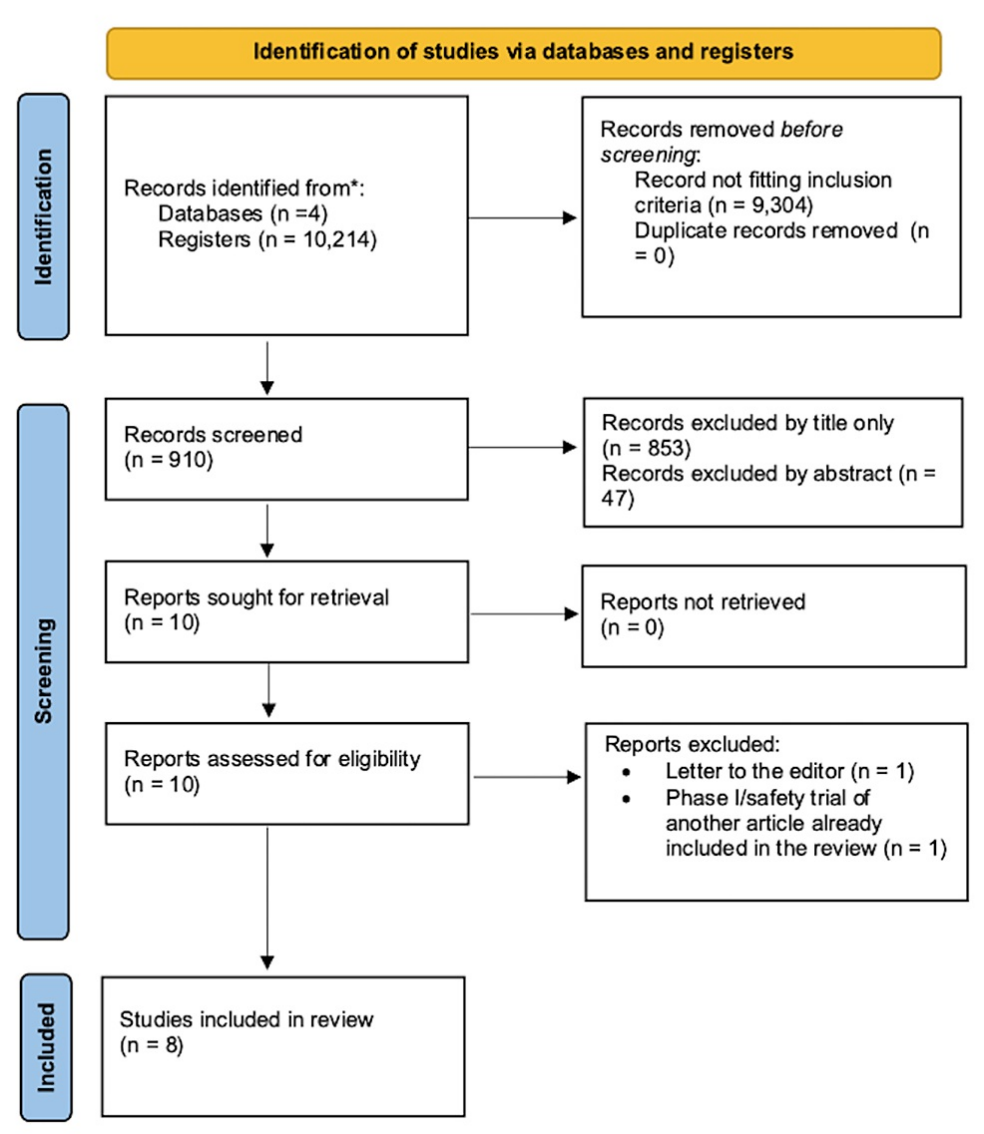
PRISMA flow diagram for article selection PRISMA: Preferred Reporting Items for Systematic Reviews and Meta-Analyses

The primary author reviewed each publication retrieved from the above database search by title and abstract and excluded all irrelevant results. Both the primary and secondary authors reviewed all subsequent publications by screening the full text and using certified critical appraisal tools: the Cochrane bias assessment tool for randomized controlled trials (RCT), the JB check tool for case reports and case series, and the methodological index for non-randomized studies (MINORS) for non-randomized safety and feasibility studies. The PRISMA flow chart can be seen in Figure 1 below, outlining the screening process.

Results

Included Studies and Characteristics

We identified 10,214 studies with the search strategy previously mentioned in four databases. After applying the previously mentioned exclusion criteria, 9304 studies were removed. There were no duplicates removed. After the title and abstract review, a further 900 studies were excluded as they did not fit the inclusion criteria previously outlined. The primary and secondary authors reviewed 10 studies and excluded an additional two studies: one study was a letter to the editor and therefore not fitting with the inclusion/exclusion criteria, and the other study was a safety and feasibility trial for an RCT that was included in the review [17].

Finally, we included eight studies: one RCT with 33 participants in the treatment group, three phase I safety and feasibility trials, and four case reports/case series. A total of 61 very premature infants (born < 32 weeks of gestation) were included in the review, of which 36 were males and 25 were females. The study description and baseline characteristics are described in Table 3.

**Table 3:** Study characteristics RCT: Randomized controlled trial; GA: Gestational age, BW: Birth weight; CA: Corrected age * [19] one patient in this study was born at 34 weeks with a birth weight of 2400 grams, was of the male sex, and given treatment at 173 days; this patient has not been included in the final results and discussion of this systematic review.

Study	Year	Type of Study	Number of patients included in the study	GA of patients	BW of patients (in grams)	Male:Female ratio	Age at the time of treatment
Ahn et al. [17]	2021	RCT	33	23-28	500-1250	17:16	11.8 ± 2 days
Lim et al. [18]	2018	Phase I / Safety Trial	6	24-28	450-990	5:1	59–187 days (average 89 days)
Nguyen et al. [19]	2020	Phase I / Safety Trial	3 *	24-28 *	650-1400 *	0:3	144–160 days
Xia et al. [9]	2022	Phase I / Safety Trial	13	27-30	850-1500	12:1	Not specified
Liem et al. [11]	2017	Case Report	1	30	1500	1:0	111 days
Lin et al. [20]	2018	Case Report	1	25	778	0:1	6 months CA
Alvarez-Fuente et al. [15]	2018	Case Report	2	24	695-700	0:2	Patient 1: 85 days, Patient 2: 150 days
Oktem et al. [21]	2020	Case Report	2 (twins)	26	750-930	1:1	32 days

The studies included in this review were conducted in Spain [15], China [9], South Korea [17], Australia [18], Vietnam [11, 19], Taiwan [20], and Turkey [21]. All studies excluded patients with congenital anomalies, septic shock or active infection, and severe intraventricular hemorrhage (> grade three), or severe brain injury. 

Most studies included infants born very prematurely with a gestation age < 32 weeks; one study [19] included four infants, of which three were born < 32 weeks of gestation while the fourth patient was born at 34 weeks of gestation. However, after careful analysis, the authors decided to include this study in the review as 75% of the patients fit the inclusion criteria of this review but the patient born at 34 weeks has not been included in the final results and analysis.

All infants were born with a birth weight of less than 1500 grams. The age of administration of the MSCs differed between studies. However, the majority of studies administered the treatment after a diagnosis of BPD had been performed as per the definition mentioned above [7]; one study [17] administered MSCs treatment prior to the development of BPD in patients on continuous invasive ventilator support and with ongoing respiratory deterioration and evolving BPD.

Risk of Bias

No studies included in this review were classified as "high risk" of bias. However, one study was a double-blinded trial with a control group for comparison of effects [17].

Outcomes

There were two crucial primary outcomes of this review: safety and efficacy.

To analyze whether the administration of MSCs is safe, we searched for any report of acute adverse events occurring during and after the administration of MSCs. Mortality within 24 hours of treatment was considered a severe adverse event most likely secondary to the treatment itself given the proximity to treatment administration. However, mortality beyond 24 hours of treatment required disclosure as to the presumed cause of death.

To conclude on the efficacy of MSCs on evolving and established BPD, the authors looked at the form of respiratory support at the time of treatment administration and on discharge, as it is known that patients with BPD are less likely to be discharged home on no respiratory support [22] and therefore, a trend towards less support would indicate that MSCs treatment for evolving and established BPD is a promising option; to corroborate this finding we also looked at the patient's age at discharge from hospital or length of hospital stay and the age at which the patient was free of all respiratory support.

Discussion

This systematic review differs from previous reviews in that it includes all studies performed in humans using MSCs to treat evolving and established BPD; this includes RCT, quasi-RCT, phase I safety and feasibility trials, and case reports. A previous systematic review published in November 2017 included only RCT and quasi-RCT [23]. Unfortunately, at the time of their review, no studies fit the inclusion criteria. Therefore, by expanding our inclusion criteria to include other types of studies, we were able to have more studies and more patients who received MSCs for evolving or established BPD.

Mesenchymal Stem Cells (MSCs) Administration

Studies included in this review differed significantly in the source of the MSCs: five studies used donor MSCs [9,17,18,19,21], while three studies used MSCs previously obtained from the patient [11,15,20]. Most studies used only one route of administration with the ratio of intravenous to intratracheal administration being 4:3. However, one study administered MSCs via both intravenous and intratracheal routes at different doses [21]. Regarding the dose of MSCs administered, the majority of studies administered a fixed dose previously agreed upon before the commencement of the study; one study was a dose escalation trial to determine the maximum tolerated dose [9], while another study [15] used data from previous trials and also data from the use of MSCs for other pediatric indications and therefore in this study, different patients received different doses. The number of administrations of MSCs also differed between studies with a ratio of 1:1 between single and multiple administrations.

Table 4 outlines the type of MSCs used in each study, the route of administration, and the dosage.

**Table 4:** Characteristics of MSCs treatment MSCs: Mesenchymal stem cells, IV: Intravenous; IT: Intratracheal

Study	Year	Source of MSCs	Route	Number of administrations	Dose
Ahn et al. [17]	2021	Allogeneic Umbilical Cord	Intratracheal	1	1X10^7^ cells/kg
Lim et al. [18]	2018	Allogeneic Amnion epithelial cells	Intravenous	1	1X10^6^ cells/kg
Nguyen et al. [19]	2020	Allogeneic Umbilical Cord	Intravenous	2 (7 days apart)	1X10^6^ cells/kg
Xia et al. [9]	2022	Allogeneic Umbilical Cord	Intravenous	1	6 patients received 1x10^6^ cells/kg and 7 patients received 5x10^6^cells/kg
Liem et al. [11]	2017	Autologous Bone Marrow	Intratracheal (last dose nebulized)	4 (30 minutes apart)	620 x 10^6^ cells
Lin et al. [20]	2018	Autologous Bone Marrow	Intratracheal	1	6.25 x 10^6^ cells/kg
Alvarez-Fuente et al. [15]	2018	Autologous Bone Marrow	Intravenous	Multiple	Patient 1 received an increasing weekly dose of 1.1 million cells/kg up to 13.9 million cells/kg while patient 2 received a fixed dose of 5 million cells/kg per week for 3 weeks
Oktem et al. [21]	2020	Allogeneic Umbilical Cord	Intravenous and Intratracheal	2 (one IV and one IT)	IV dose was 2 x 10^6^ cells/kg and IT dose was 1 x 10^7^cells/kg

Safety of MSCs Administration

Most studies reported the presence or absence of adverse effects during and in the immediate period of MSCs administration. One study reported the development of a transient cardiorespiratory compromise during manual intravenous MSCs infusion, which was characterized by sudden acute hypoxia and bradycardia without changes in blood pressure [18]; this was later corrected for further patients in the same study by changing the administration protocol to include a 30-minute infusion via syringe-driver with a transfusion filter, and further diluting the preparation. Another study reported the onset of tracheal obstruction episodes with intratracheal administration of MSCs; these episodes were characterized by cyanosis with reduced oxygen saturations and bradycardia requiring immediate tracheal suctioning and a switch to nebulized MSCs administration [11].

Regarding mortality, there were no reported deaths within 24 hours of MSCs administration. Four studies reported deaths occurring more than 24 hours after MSCs administration and before patient discharge home [9,15,18,21]. However, after careful analysis, the authors of each study concluded that the deaths were not treatment-related: one patient died one month after treatment due to accidental extubation resulting in multiorgan failure [18]; another study reported deaths on both patients included, however, their case series involved patients with severe BPD and where MSCs were administered as an experimental treatment after failure of all other therapies and with parental knowledge and consent [15]; the third study administered MSCs in premature twins born at 26 weeks of gestation and reported death in the female twin with lower birth weight who remained on mechanical ventilation throughout the admission and unable to wean off respiratory support [21]. The final study did not report the cause of death of the two patients, however, the authors declared a detailed review and conclusion that the two deaths were not treatment-related [9].

Table 5 outlines the safety description of each study.

**Table 5:** Safety outcomes MSCs: Mesenchymal stem cells, N/A: Non-applicable

Study	Year	Adverse events reported	Death within 24 hours of administration	Death pre-discharge
Ahn et al. [17]	2021	Not reported in this paper but reported in the phase I safety trial [24]	N/A	N/A
Lim et al. [18]	2018	1 infant suffered a transient cardiorespiratory compromise	0	1
Nguyen et al. [19]	2020	No adverse events	0	0
Xia et al. [9]	2022	No adverse events	0	2 (patient 1 died on day 10 post-infusion and patient 2 on day 24 post-infusion)
Liem et al. [11]	2017	Episodes of tracheal obstruction after administration of MSCs	0	0
Lin et al. [20]	2018	Not documented	0	0
Alvarez-Fuente et al. [15]	2018	No adverse events	0	2 (one patient died 3 weeks post-infusion and the other patient died 6 weeks post-infusion)
Oktem et al. [21]	2020	Not documented	0	1 (female patient died on day 18 post-treatment)

Efficacy of MSCs in the Treatment of BPD

The majority of patients included in this review were on invasive mechanical ventilation before treatment administration (n=42) and therefore considered to have severe BPD as per the classification [7], with six patients remaining on continuous positive airway pressure (CPAP) ventilation and 11 patients on low flow nasal cannula oxygen. 

The method of respiratory support at discharge is not documented in two studies. Interestingly, all surviving patients on invasive ventilatory support at the time of treatment administration were discharged home on some respiratory support either in the form of low-flow nasal cannula oxygen [18], or with oxygen via tracheostomy [20]. Only four studies reported the age of patients at the time of discharge, with most patients remaining in the hospital between 80 to 174 days, which signifies a corrected age at discharge between term age and three months; one study [18] had two patients remaining in the hospital for more extended periods (238 days and 388 days; corrected age at discharge: five and 11 months, respectively).

The age at which patients were free from any respiratory support varies and is only documented in three studies [11,18,19]. However, on-premise patients remained on low-flow nasal cannula oxygen for a few months after discharge home (minimum two months and maximum 33 months corrected age).

Table 6 summarises the efficacy outcomes of each study.

**Table 6:** Efficacy outcomes CA: Corrected age, SVRA: Self ventilating on room air, IMV: Invasive mechanical ventilation, sBPD: Severe bronchopulmonary dysplasia, CPAP: Continuous positive airway pressure, O2: Oxygen, mBPD: Moderate BPD, N/A: Non-applicable, RIP: Rest in peace/died *Some of the results in this table were obtained from the two-year follow-up paper published by the same group [25]

Study	Year	Respiratory support at the time of treatment	Duration of hospital stay/CA at discharge	Respiratory support at discharge	Age when SVRA
Ahn et al. [17]	2021	IMV	108 ± 28 days	Not documented	Not documented
Lim et al. [18]	2018	3 patients on IMV (sBPD) and 3 patients on CPAP (sBPD)	155-388 days (CA 7 weeks-11 months)	Nasal cannula O2 (mBPD)	9-33 months CA
Nguyen et al. [19]	2020	Nasal cannula O2 (mBPD)	154-174 days (CA 6-13 weeks)	2 patients were SVRA (mild BPD) and 1 patient on nasal cannula O2 (mBPD)	1-3 months CA
Xia et al. [9]	2022	4 patients on IMV (sBPD), 3 patients on CPAP (sBPD), and 6 patients on nasal cannula O2 (mBPD)	Not documented	Not documented	Not documented
Liem et al. [11]	2017	Nasal cannula O2 (mBPD)	129 days (CA 8 weeks)	SVRA (mild BPD)	5 months CA
Lin et al. [20]	2018	IMV (sBPD)	N/A (admitted from home)	Oxygen via tracheostomy (mBPD)	Not documented
Alvarez-Fuente et al. [15]	2018	Not documented	N/A (RIP)	N/A	N/A
Oktem et al. [21]	2020	1 patient on IMV (sBPD) and 1 patient on nasal cannula O2 (mBPD)	Not documented	1 patient RIP and 1 patient was SVRA (mild BPD)	1 patient RIP and 1 patient on discharge (age not documented)

Another point to note is that the only trial that compared the use of MSCs versus placebo with a control group [17] reported no statistical difference between both groups in the duration of invasive mechanical ventilation, CPAP, total ventilator days, and duration of oxygen therapy. It also reported no statistical difference in the length of hospital stay between both groups. A further subgroup analysis performed by this study group reviewed the same outcomes in the 23 to 24 weeks of gestation and the 25 to 28 weeks of gestation groups; it concluded after Bayesian analysis that the incidence of severe BPD or death was reduced in patients that received MSCs treatment in the younger gestational group in comparison to the older group, however, the authors disclosed that the study was underpowered to detect this as statistically significant.

Limitations

Though our study has several strengths, there were certain limitations essential to note. Firstly, only one RCT was included and the majority were case reports, this is due to the lack of completed RCTs in this subject. Secondly, a patient included in one of the studies [19] had to be excluded from the final results and analysis as this patient did not fulfill the inclusion criteria of very premature birth (<32 weeks). However, given that the remaining three patients fulfilled the inclusion criteria and showed some signs of improvement by becoming free of oxygen soon after MSCs therapy, the authors made the decision to include three out of the four patients of this study. Finally, our search strategy excluded studies not published in English and where the full text was not available for free; the inclusion of these might have expanded the literature research and given a better picture.

## Conclusions

The administration of MSCs appears to be both safe and feasible according to initial data, however, its effectiveness is not yet certain. The lack of well-powered studies remains a big deficit in the field of MSCs therapy for premature neonates as can be seen by the evidence presented in this review which consists primarily of case reports. Most patients showed some signs of improvement post-treatment, however, they still required respiratory support on discharge and were subsequently weaned off which is the typical course of the disease. Therefore, it is not possible to establish whether the use of MSCs was of benefit. Only one study was an RCT and this did not show any statistically significant benefit of MSCs versus placebo. Interestingly, further analysis revealed a possible benefit when the treatment was applied to a younger gestational age (23 to 24 weeks), however, the study was underpowered to detect this. Given the current knowledge of the benefit of MSCs in the treatment of other conditions, further research should focus on the management of conditions with a high burden of disability such as the ones that affect premature infants. Research on the prevention and treatment of BPD should not be halted as this is a severely debilitating disease of prematurity with significant costs to the patient, family, and hospital institutions.
